# PTTG1 induces pancreatic cancer cell proliferation and promotes aerobic glycolysis by regulating c-myc

**DOI:** 10.1515/biol-2022-0813

**Published:** 2024-02-08

**Authors:** Yong Wang, Jianping Hu, Chen Chen, Yongbo Li

**Affiliations:** Department of General Surgery, Suqian First Hospital, No. 120 Suzhi Road, Sucheng District, Suqian, Jiangsu Province, 223800, China

**Keywords:** aerobic glycolysis, cell proliferation, c-myc, PTTG1, pancreatic cancer

## Abstract

This study aimed to clarify the role of *pituitary tumor-transforming gene 1* (PTTG1) in proliferation, migration, invasion, and aerobic glycolysis of pancreatic cancer cells, and evaluate the potential of PTTG1 as a therapeutic target. PTTG1 expression in pancreatic cancers was analyzed using the GEPIA databank. In the Panc1 cell with the PTTG1 knockdown or Mia-PaCa2 cells with PTTG1 overexpression, the cell proliferation was evaluated using cell viability curves and colony formation, and wound heal assay and transwell assay were performed to evaluate the migration and invasion, respectively. Furthermore, a western blot was performed to evaluate the expressions of PTTG1, proliferating cell nuclear antigen, E-cadherin, N-cadherin, and c-myc. Meanwhile, the glucose uptake, extracellular acidification rates (ECAR), and oxygen consumption rates (OCR) were analyzed. Our results showed that PTTG1 expression is upregulated in pancreatic cancer, which promoted cell proliferation. Low PTTG1 contributed to higher disease-free survival and overall survival. In Panc1 cell, PTTG1 knockdown resulted in reduced cell viability and colony formation. The migration and invasion abilities of the cells were also reduced in Panc1 with PTTG1 knockdown. Correspondingly, PTTG1 knockdown decreased c-myc expression, glucose uptake, ECAR, and OCR in Panc1 cells. In Mia-PaCa2 cells, PTTG1 overexpression promoted cell proliferation, aerobic glycolysis, and translocation of β-catenin to the nucleus by regulating c-myc. In conclusion, PTTG1 induces proliferation, migration, and invasion, and promotes aerobic glycolysis in pancreatic cancer cells via regulating c-myc, demonstrating the potential of PTTG1 as a therapeutic target.

## 1 Introduction

Pancreatic ductal adenocarcinoma (PDAC) is an aggressive and lethal solid tumor with a poor prognosis [1]. PDAC is sixth and seventh the leading cause of cancer-related deaths in men and women, respectively [2]. Therefore, elucidating the pathogenesis of PDAC and developing effective therapeutic targets are of great significance for pancreatic cancer treatment.

Aerobic glycolysis is a metabolic process, in which cancer cells generate energy by converting glucose into lactate. This metabolic pathway is often upregulated in cancer cells and is known to promote cell proliferation and survival. Lactate dehydrogenase is a valuable enzyme for assessing cancer patients due to its sensitivity to cellular metabolic state, glycolytic direction, and potential for targeted therapy based on altered metabolism [3]. The mutation or overexpression of c-myc frequently contributes to cell cycle arrest or tumor growth [4]. Studies have reported that knockdown of *pituitary tumor-transforming gene 1* (PTTG1) can downregulate c-myc-mediated aerobic glycolysis in cancer cells. PTTG1 can interact with c-myc and enhance its transcriptional activity, leading to the upregulation of glycolytic enzymes [5,6,7].

PTTG1 has been shown to be aberrantly expressed in several tumors such as breast cancer, esophageal cancer, and adrenocortical carcinoma [8,9,10]. PTTG1 regulates the G1/S cell cycle process and inhibits cell angiogenesis in glioma cells [11,12]. PTTG1 was found to be an inflammation-related oncogene that can induce c-myc [13]. PTTG1 is overexpressed in human pancreatic cancer tissues [14], but its role in pancreatic cancer is still unclear.

This study showed that PTTG1 is upregulated in pancreatic cancer. Panc1 cells with PTTG1 knockdown and Mia-PaCa2 cells with PTTG1 overexpression were used to evaluate the proliferation, migration, invasion, and aerobic glycolysis of pancreatic cancer. The results demonstrated that PTTG1 induces proliferation and aerobic glycolysis through regulating c-myc.

## 2 Materials and methods

### 2.1 Cell culture

PDAC cell line HPNE (CRL-4023, ATCC) and human pancreatic cancer cell lines including Sw1990 (CRL-2172, ATCC), Capan1 (HTB-79, ATCC), Panc1 (CRL-1469, ATCC), and Mia-PaCa2 (CRL-1420, ATCC) were maintained in Dulbecco’s modified Eagle’s medium (DMEM) (Gibco, NY, USA) added with 10% fetal bovine serum (FBS) (Gibco) in a 37°C incubator supplied with 5% CO_2_.

### 2.2 Bioinformatic analysis

The analysis of PTTG1 expression was performed in pancreatic cancer tissues via an online tool (http://gepia.cancer-pku.cn/).

PTTG1 overexpression and knockdown are explained in this section. The coding sequence of PTTG1 was amplified using primers (forward: 5′- ATCGGATCCACCATGGCTACTCTGATC-3′; reverse: 5′-ACAGTCGACTTAAATATCTATGTCA-3′), which was cloned into pcDNA3.1 vector using BamH I and Sal I sites. The acquired plasmid named pc-PTTG1 was used for PTTG1 overexpression, and the pcDNA3.1 empty vector was taken as a negative control. For PTTG1 knockdown, two PTTG1 siRNA (target sequence: GACCTGCAATAATCCAGAA and GGCTACTCTGATCTATGTT) and the negative control siRNA (siNC, target sequence: TCCTTCGAACGTGCTACGT) were synthesized from Genepharma (Shanghai, China). The 50 nM siRNA was transfected according to the product instructions.

### 2.3 Cell viability assay

Panc1 or Mia-PaCa2 cells were cultured in 96-well plates followed by PTTG1 knockdown or overexpression, respectively. Twenty-four hours later, the cells were replaced with a new medium supplemented with 10 μl CCK-8 (CA1210; Solarbio, Beijing, China) per well and cultured for another 5 days. On each day, the absorbance was analyzed at 450 nm wavelength, and the cell viability ratio was calculated.

### 2.4 Colony formation assay

Panc1 or Mia-PaCa2 cells were seeded into six-well plates with about 500 cells each well. Each well was transfected with siRNAs as described in the cell proliferation assay. When the ocular cell clusters were formed, the cells were fixed in 4% paraformaldehyde and stained with 0.1% violet crystal (Cat No. 548-62-9, Sigma-Aldrich, Shanghai, China). The visible colonies were counted, and the survival curves were made by GraphPad Prism 8.

### 2.5 Wound heal assay

Panc1 or Mia-PaCa2 cells were seeded into a six-well plate, after the cells were attached to the bottom, a pipet tip was used to scratch the layer of cells as described previously [15]. Photos were taken immediately, and widths of the scratch line were measured using ImageJ at 24 h later.

### 2.6 Transwell assay

Panc1 or Mia-PaCa2 cells were resuspended in serum-free medium and plated into the upper compartment of a transwell chamber with 8.0 μm pore polycarbonate membrane inserts (CLS3422-48EA, Corning, USA), and the lower chamber was supplemented with media containing 10% FBS. Then the cells that passed through the polycarbonate membrane were stained with 0.1% crystal violet. For each sample, more than 10 fields were selected randomly, and the mean number of stained cells was counted.

### 2.7 Western blot

The total protein of Panc1 or Mia-PaCa2 cells was extracted using lysis buffer (89901, Thermo Scientific, Carlsbad, California, USA). The total protein concentration in the lysates was determined using the Bradford method [16]. For each sample, 40 μg protein were processed for immunoblot with primary antibodies (Table 1) at 4°C overnight and then incubated with horseradish peroxidase-conjugated goat antirabbit IgG (B900210, ProteinTech Group; 1:5,000) at room temperature for 1 h. Finally, the target bands were visualized with ECL reagents (Solarbio, Beijing, China) using the ChemiDoc Imaging system (Bio-Rad). The relative intensity was measured by ImageJ software and normalized to GAPDH.

**Table 1:** Antibodies information

Protein	Cat. no.	Manufacturer	Dilution
PTTG1	13445	Cell Signaling Technology, MA, USA	1:1,000
PCNA	Ab18197	Abcam, Cambridge, MA, USA	1:2,000
N-cadherin	22018-1-AP	Proteintech Group, Rosemont, IL, USA	1:5,000
E-cadherin	20874-1-AP	Proteintech Group, Rosemont, IL, USA	1:20,000
c-myc	Ab32072	Abcam, Cambridge, MA, USA	1:3,000
GAPDH	10194-1-AP	ProteinTech Group, Rosemont, IL, USA	1:8,000

### 2.8 Glucose consumption

Panc1 or Mia-PaCa2 cells were seeded into 96-well plates supplemented with 100 μl DMEM. Then, 4 h later, the medium was replaced. After incubation for 24 h under 3% or 21% O_2_, the supernatant was analyzed for glucose intake using a glucose assay kit (Solarbio, Beijing, China) following the manual instruction.

### 2.9 Metabolic analyses

Seahorse XF96 Extracellular Flux Analyzer was used for metabolic analysis. Briefly, Panc1 or Mia-PaCa2 cells were plated on XF96 PET 96-well plates overnight for analyzing metabolic fluxes. The ECAR and OCR values were measured for four cycles. The extracellular acidification rates (ECAR) and oxygen consumption rates (OCR) values obtained in XF96 assays were normalized using quantification of cellular DNA. At least three repeated experiments were completed for data analysis.

### 2.10 Quantification and statistical analysis

Data are presented as mean ± standard deviation (SD) from three biological replicates. The survival analysis was conducted using the log-rank test. In addition, differences between any two groups were assessed through unpaired *t*-tests, while multiple group comparisons were analyzed using analysis of variance.

## 3 Results

### 3.1 PTTG1 is highly expressed in pancreatic cancer

**Figure 1:**
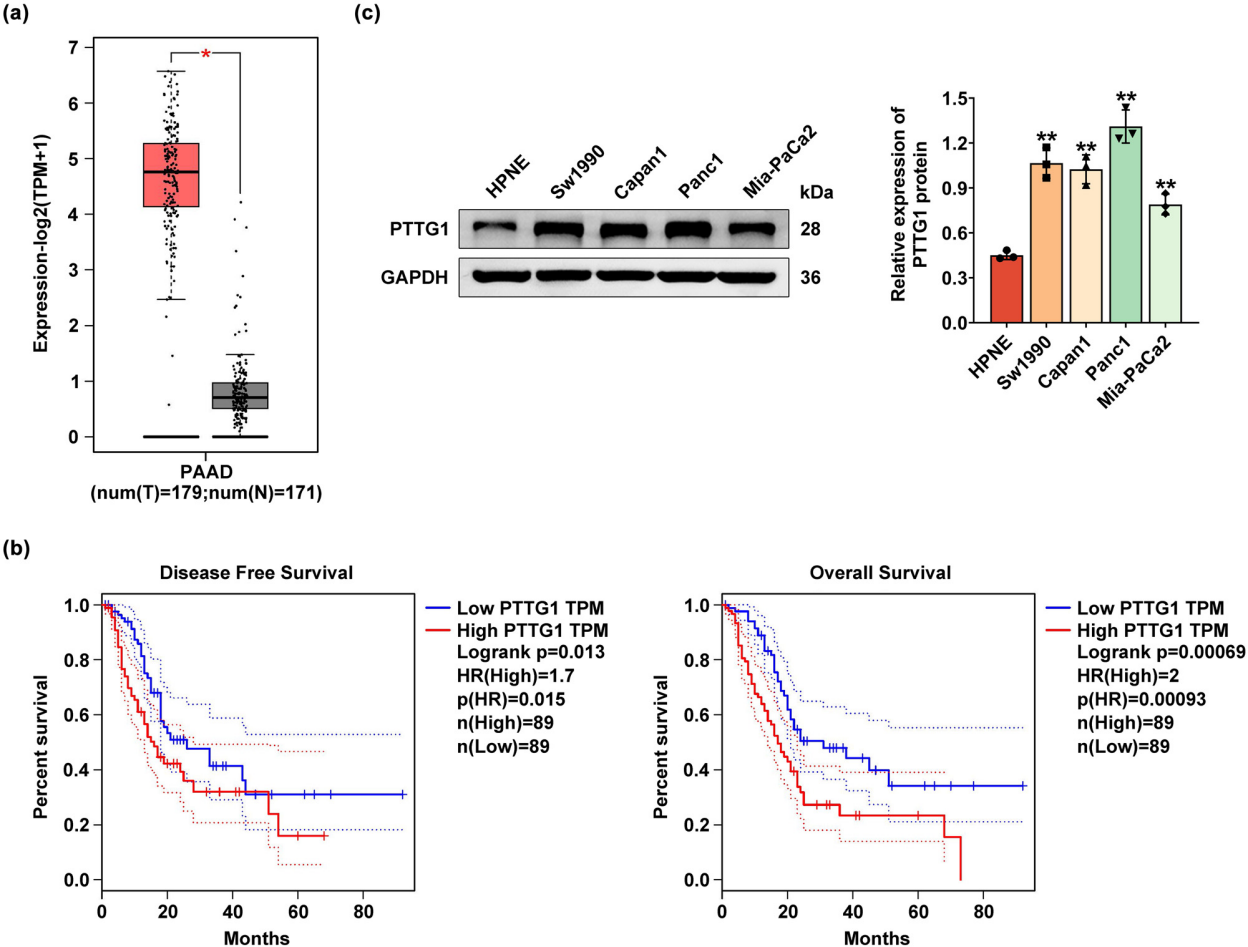
PTTG1 is highly expressed in pancreatic cancer. (a) PTTG1 expression levels were analyzed using the GEPIA website. red, pancreatic adenocarcinoma tissues (*n* = 179); gray, normal tissues (*n* = 171). (b) Western blot was performed to analyze PTTG1 expression in pancreatic cancer cells including Sw1990, Capan1, Panc1, Mia-PaCa2 and PDAC cell lines HPNE. (c) The high expression of PTTG1 was associated with poorer overall survival and disease-free survival in patients with pancreatic cancer (*n* = 89). The data from three repeated experiments were used for the statistical analysis. Error bar, mean ± SD; *p < 0.05, **p < 0.01.

PTTG1 has been reported to be overexpressed in several cancers, so we examined the PTTG1 expression in pancreatic cancer. The analysis of PTTG1 expression levels using the GEPIA website revealed that PTTG1 was indeed highly expressed in pancreatic cancer tissues. The box plot generated from the analysis showed a significant increase in PTTG1 expression levels in pancreatic adenocarcinoma tissues (red) compared to matched normal tissues (gray) (Figure 1a). Western blot results indicated that PTTG1 expression in pancreatic cancer cells including Sw1990, Capan1, Panc1, and Mia-PaCa2 is significantly higher than that in HPNE, which is the normal cell line (Figure 1b). These data demonstrated that PTTG1 is highly expressed in pancreatic cancer.

Then, the effect of PTTG1 on pancreatic cancer progression was investigated, and the high expression of PTTG1 was linked to poorer overall survival and disease-free survival in patients with pancreatic cancer (Figure 1c). Thus, PTTG1 is highly expressed in pancreatic cancer tissues, which is negatively associated with poor prognosis.

### 3.2 PTTG1 promotes proliferation of pancreatic cancer cells

**Figure 2:**
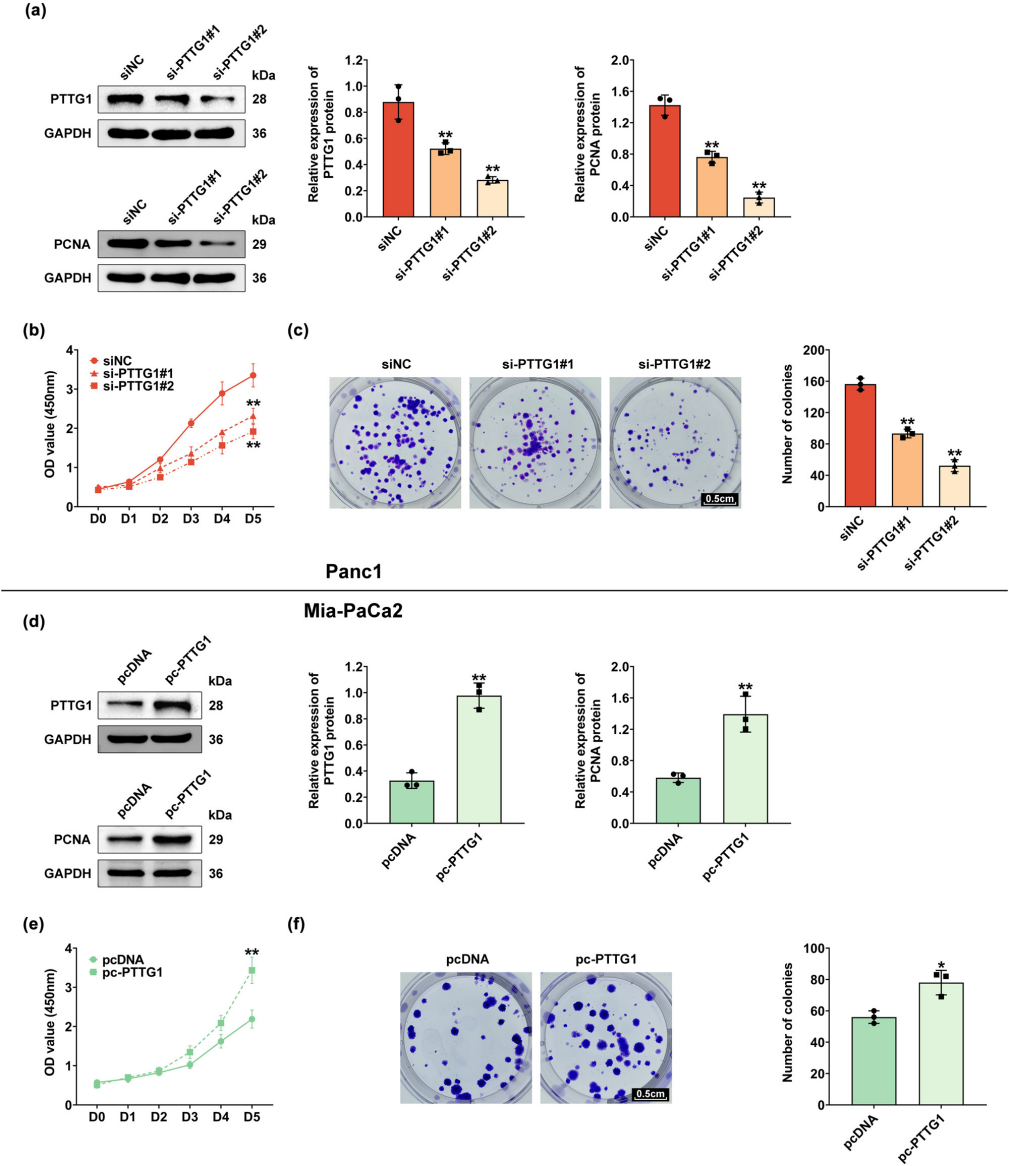
PTTG1 promotes proliferation of pancreatic cancer cells. (a) Western blot was used to measure the protein level of PTTG1 and PCNA in panc1 cells transfected with si-PTTG1 #1 and #2. (b) Cell proliferation curves of Panc1 with PTTG1 knockdown using si-PTTG1 #1 and #2. (c) Clone formation of Panc1 cells with PTTG1 knockdown using si-PTTG1 #1 and #2. (d) Western blot was used to measure the protein level of PTTG1 and PCNA in Mia-Paca2 cells with PTTG1 overexpression. (e) Cell proliferation curves of Mia-Paca2 with PTTG1 overexpression. (f) Clone formation of Mia-Paca2 cells with PTTG1 overexpression. All the experiments were repeated at least three times. Error bar, mean ± SD; *p < 0.05, **p < 0.01.

Highly expressed PTTG1 is implicated in cancer progression. To study the effect of PTTG1 on the proliferation of pancreatic cancer cells, siRNA-mediated knockdown of PTTG1 was performed in Panc1 cells with transfection of si-PTTG1 #1 and #2. Western blot results showed that both si-RNAs significantly declined PTTG1 expression, and proliferating cell nuclear antigen (PCNA) expression was also significantly reduced in si-PTTG1 #1 and #2-inhibited cells (Figure 2a). PTTG1 knockdown using si-PTTG1 #1 and #2 reduced the cell proliferation (Figure 2b) and colony formation (Figure 2c). Therefore, knockdown of PTTG1 inhibits the proliferation of pancreatic cancer cells.

Overexpression of PTTG1 was performed with transfection of pc-PTTG1 into Mia-PaCa2 cells. Western blot data revealed that the protein levels of PTTG1 and PCNA were elevated compared to that of control (pcNDA) (Figure 2d). PTTG1 overexpression significantly increased cell proliferation (Figure 2e) and colony formation (Figure 2f). Therefore, altogether, these results suggested that PTTG1 overexpression promotes the proliferation of pancreatic cancer cells.

### 3.3 PTTG1 promotes migration and invasion of pancreatic cancer cells

**Figure 3:**
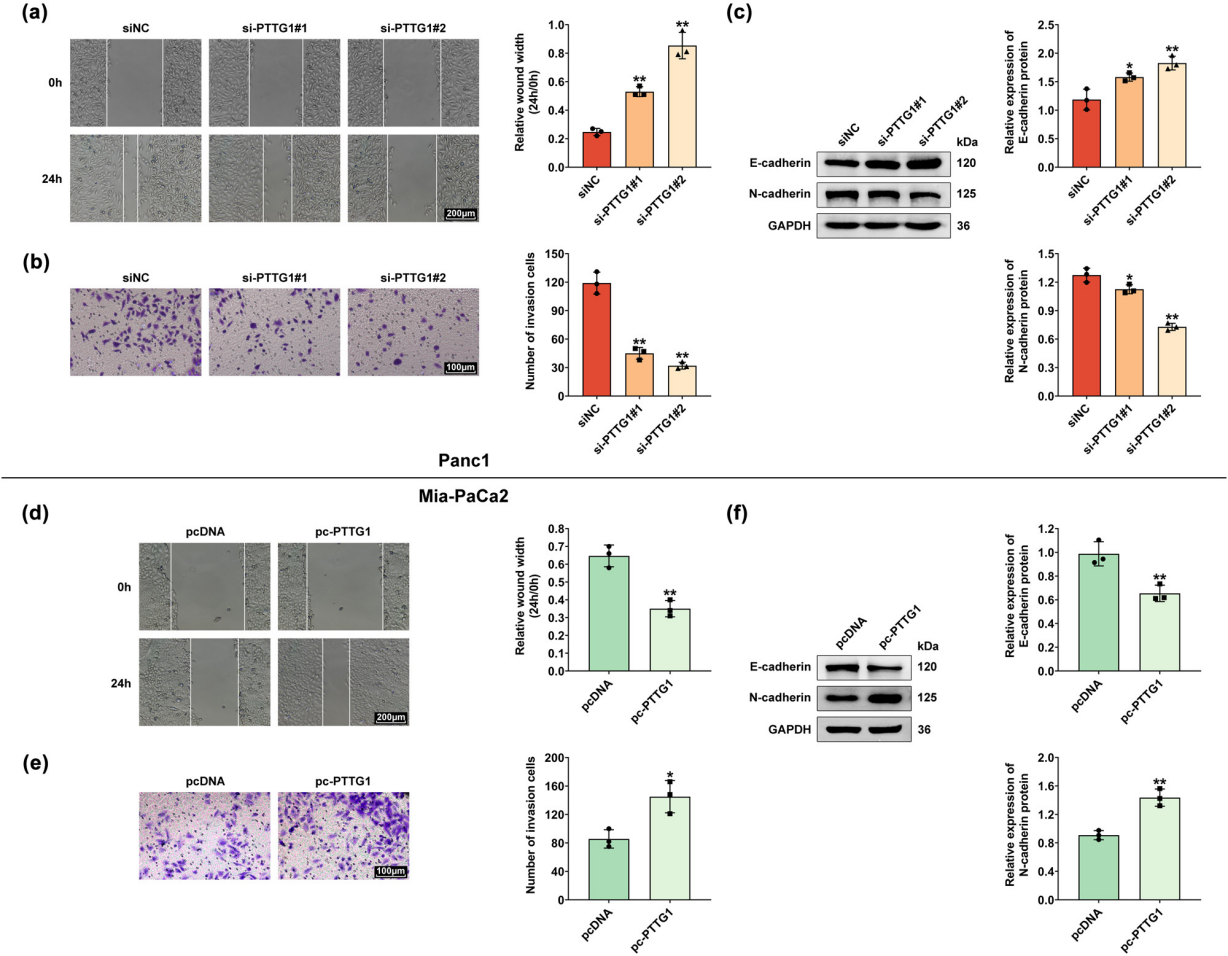
PTTG1 promotes migration and invasion of pancreatic cancer cells. (a) Wound healing assay was performed to detect migration in Panc1 cells with PTTG1 knockdown. (b) The transwell assay was performed to analyze the invasion of Panc1 cells with PTTG1 knockdown. (c) Western blot was performed to evaluate the expression of E-cadherin and N-cadherin in Panc1 cells. (d) Wound heal assay was performed to detect migration in Mia-Paca2 cells with PTTG1 overexpression. (e) The transwell assay was performed to analyze the invasion of Mia-Paca2 cells with PTTG1 overexpression. (f) Western blot was performed to evaluate the expression of E-cadherin and N-cadherin in in Mia-Paca2 cells. Three repeated experiments were used for analysis. Error bar, mean ± SD; *p < 0.05, **p < 0.01.

Migration and invasion are critical processes involved in cancer progression and metastasis. To clarify whether PTTG1 regulates the migration and invasion of pancreatic cancer, wound-healing assay was performed to detect cell migration of Panc1 cells. The results showed that the wound width of si-PTTG1 transfected cells was larger than the control group, indicating that the cell migration ability was significantly reduced in Panc1 cells transfected with si-PTTG1 #1 or #2 (Figure 3a). The transwell assays demonstrated that PTTG1 knockdown by transfection with si-PTTG1 #1 or #2 reduced the invasion of Panc1 cells (Figure 3b). Furthermore, western blot was performed to evaluate the expression of E-cadherin and N-cadherin. The expression level of E-cadherin was significantly increased in PTTG1 knockdown cells, while N-cadherin expression was significantly declined when PTTG1 was knockdown (Figure 3c). The aforementioned data suggested that PTTG1 knockdown attenuates the migration and invasion of pancreatic cancer cells.

PTTG1 overexpression in Mia-Paca2 cells promoted cell migration (Figure 3d), which was demonstrated by smaller wound width when PTTG1 was overexpressed by pc-PTTG1 transfection. Similarly, PTTG1 overexpression increased the number of invasion cells and the number of colony formation (Figure 3e). The expression level of E-cadherin was reduced, while N-cadherin expression was elevated when PTTG1 was overexpressed (Figure 3f). In summary, overexpression of PTTG1 accelerates migration and invasion of pancreatic cancer cells.

### 3.4 PTTG1 regulates c-myc-mediated aerobic glycolysis

**Figure 4:**
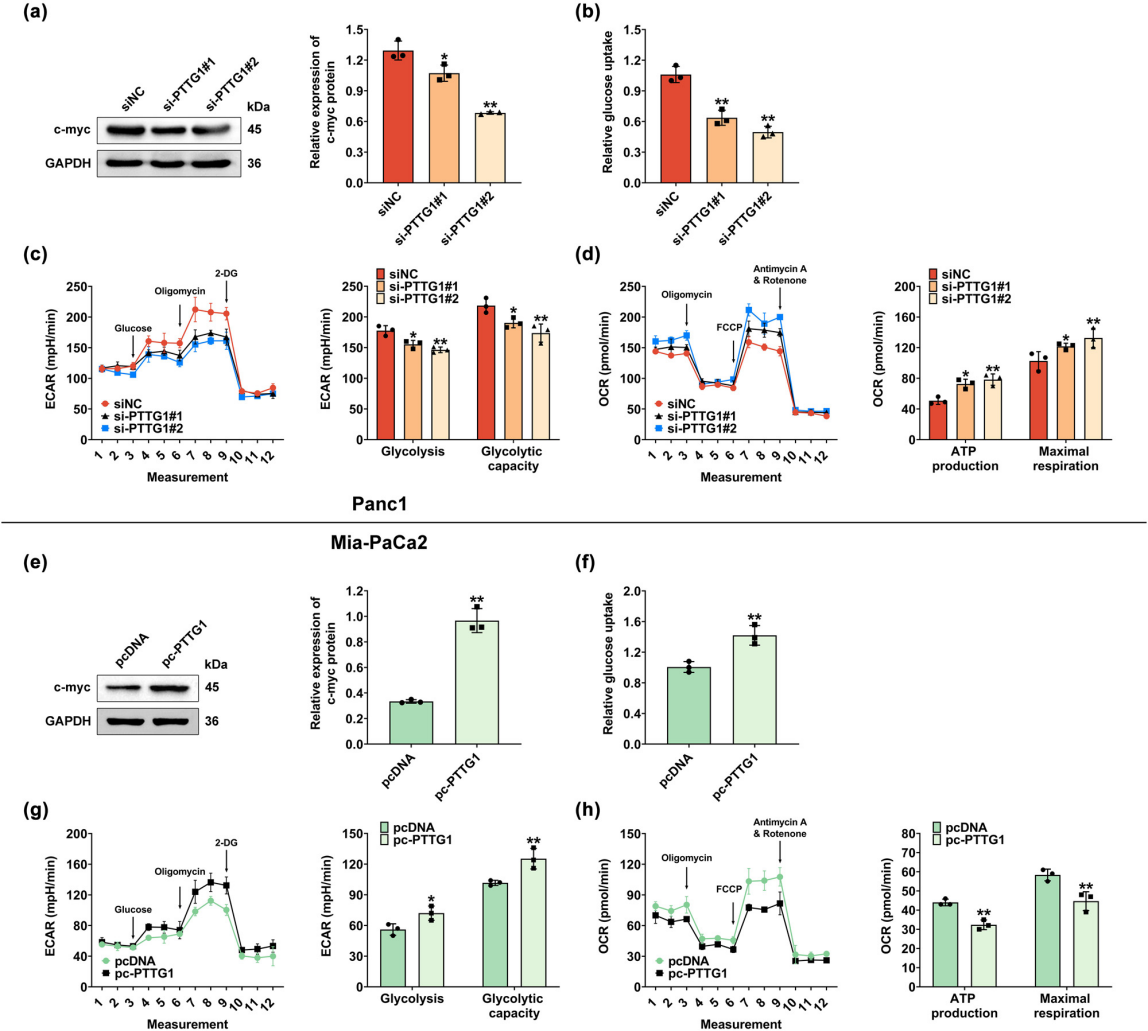
PTTG1 regulates c-myc mediated aerobic glycolysis. (a) Western blot was used to evaluate the c-myc expression in Panc1 cells with PTTG1 knockdown. (b) PTTG1 knockdown significantly reduced the glucose uptake. (c) ECAR was measured using XF96 Flux analyzer to detect changes in the extracellular acidification rate of Panc1 cells with PTTG1 knockdown. (d) OCR was analyzed to measure the ATP production and maximal respiration in Panc1 cells with PTTG knockdown. (e) c-myc expression was measured using western blot in Mia-PaCa2 cells with PTTG1 overexpression. (f) PTTG1 overexpression promoted glucose uptake in Mia-PaCa2 cells. (g) ECAR was measured to detect changes in the extracellular acidification rate of Mia-PaCa2 cells with PTTG1 overexpression. (h) OCR was analyzed to measure the ATP production and maximal respiration in Mia-PaCa2 cells with PTTG overexpression. The data from three independent replications were used for the analysis. Error bar, mean ± SD; *p < 0.05, **p < 0.01.

PTTG1 was found to be an inflammation-involved oncogene by inducing c-myc. To investigate whether PTTG1 regulates c-myc-mediated aerobic glycolysis in pancreatic cancer cells, western blot was conducted to evaluate the c-myc expression in Panc1 cells transfected with si-RNA PTTG1. The results indicated that PTTG1 knockdown significantly declined c-myc expression (Figure 4a) and reduced the glucose uptake (Figure 4b). Moreover, ECAR was measured using XF96 Flux analyzer, which detects changes in the extracellular acidification rate of cells. When glucose is added, the ECAR measurement increases, reflecting the additional acidification produced by the increasing glycolytic activity. The results in this study showed that the ECAR was decreased in si-PTTG #1 and #2 groups compared to the control group, indicating that glycolysis or glycolysis ability was markedly reduced when PTTG1 expression was downregulated (Figure 4c). Meanwhile, the OCR in si-PTTG #1 and #2 was increased, and the ATP production and maximal respiration were elevated in si-PTTG #1 and #2 groups (Figure 4d). Therefore, knockdown of PTTG1 downregulates c-myc-mediated aerobic glycolysis.

Correspondingly, PTTG1 overexpression significantly promoted c-myc expression (Figure 4e) and increased glucose uptake (Figure 4f). In addition, PTTG1 overexpression significantly increased the ECAR value, and glycolysis or glycolysis ability (Figure 4g). However, the OCR was decreased, and the ATP production and maximal respiration were repressed when PTTG1 was overexpressed (Figure 4h). Therefore, PTTG1 overexpression promoted c-myc-mediated aerobic glycolysis.

## 4 Discussion

Pancreatic cancer is threatening the public health, and developing novel molecular therapies is necessary for pancreatic cancer treatment. Surgery, chemotherapy, and radiation therapy are common methods to treat pancreatic cancer. However, the prognosis for pancreatic cancer is often poor, with a low overall survival rate. Multiple reports have demonstrated that PTTG1 expression is elevated in pancreatic cancer tissues, suggesting its involvement progression of this disease. In addition, several *in vitro* studies have shown that PTTG1 is overexpressed in pancreatic cancer cell lines, including BxPC-3, AsPC-1, and Panc-1 cells. In this study, PTTG1 was confirmed to be highly expressed in pancreatic cancers, which is consistent with the previous findings.

Accumulating studies clarified that PTTG1 overexpression is associated with poor prognosis in various types of cancers. High expression of PTTG1 was related to poor overall survival and disease-free survival in patients with pancreatic cancer. *In vitro* studies have shown that PTTG1 promotes cell cycle progression and increases the proliferation of bladder cancer cells [17]. PTTG1 can also interact with other proteins related to cell cycle regulation, such as cyclin D1 and cyclin-dependent kinases (CDK4), to enhance cell proliferation [18]. Furthermore, in vivo study using mouse models has demonstrated that PTTG1 promotes tumor growth and proliferation [19]. Inhibition of PTTG1 expression or activity can suppress the proliferation of pancreatic cancer cells, suggesting that PTTG1 may be a potential target for the development of novel anticancer therapies. In summary, overexpression of PTTG1 promotes the proliferation of pancreatic cancer cells by promoting cell cycle progression and interacting with other proteins involved in cell cycle regulation.

PTTG1 has been reported to inhibit cell angiogenesis in glioma cells and regulate the G1/S cell cycle process [11,12]. Migration and invasion are critical processes involved in cancer progression and metastasis. PTTG1 increases the migration and invasion of pancreatic cancer cells by regulating various signaling pathways. For example, PTTG1 can activate the phosphatidylinositol 3-kinase (PI3K)/protein kinase B (AKT) signaling pathway, which is crucial for cancer cell migration and invasion [20]. Moreover, PTTG1 can increase the expression of matrix metalloproteinases that degrades the extracellular matrix and allows cancer cells to invade surrounding tissues [21,22]. Moreover, in vivo studies using mouse models of pancreatic cancer have demonstrated that PTTG1 promotes tumor growth, metastasis, and invasion. PTTG1 was found to be an inflammation-involved oncogene by inducing c-myc [13]. PTTG1 can influence glucose metabolism and insulin sensitivity. It regulates metabolic pathways, potentially affecting energy balance and metabolic disorders. PTTG1 plays a crucial role in hepatocellular carcinoma development by promoting asparagine metabolism and activating the mechanistic target of rapamycin pathway, indicating the potential of PTTG1 as therapeutic and diagnostic targets [23]. This study demonstrated that PTTG1 is upregulated in pancreatic cancer, and PTTG1 expression positively regulates the proliferation, migration, invasion, and aerobic glycolysis of pancreatic cancer through regulating c-myc. Inhibition of PTTG1 expression or activity can suppress the metastasis of pancreatic cancer cells, suggesting that PTTG1 may be a potential target for the development of novel anticancer therapies. More investigations might be necessary to elucidate the role of PTTG1 in animal models.

In conclusion, overexpression of PTTG1 promotes cell proliferation, migration, and invasion of pancreatic cancer, and it also regulates c-myc-mediated aerobic glycolysis. Targeting PTTG1 may be a promising strategy for the treatment of pancreatic cancer.
